# Analysis of Multi-Physics Coupling of Small Holes in GH4169 Alloy by Electrolytic Processing of Tube Electrodes

**DOI:** 10.3390/mi12070828

**Published:** 2021-07-15

**Authors:** Zhaolong Li, Ye Dai

**Affiliations:** 1 Key Laboratory of Advanced Manufacturing Intelligent Technology of Ministry of Education, Harbin University of Science and Technology, Harbin150080, China; lizhaolong@hrbust.edu.cn; 2 School of Mechanical and Power Engineering, Harbin University of Science and Technology, Harbin 150080, China

**Keywords:** high-temperature resistant nickel-based alloy, electrolytic processing, pulsed power supply, multi-physical field coupling, histomorphology

## Abstract

This paper presents a simulation and experimental study of the structure of small holes in GH4169 alloy electrolytic ally processed by tube electrodes with different characteristic power sources. It analyzes the multi-physical field coupling relationship of flow, temperature, and electric fields within the interstitial space. The results indicate that the tube electrode electrolytic processing of the GH4169 alloy small hole structure with a pulsed power supply has more uniform temperature and current density distribution within the gap, which is beneficial to the processing accuracy and smoothness of the small hole structure. Meanwhile, SEM was used to analyze the microscopic morphology of the electrode end surface during short-circuiting, and it was concluded that as the processing continued, the electrode end surface gradually produced a non-metallic oxide layer, which destroyed the electric field of the gap and affected the processing stability. The use of high-frequency positive and negative pulse power can effectively avoid the generation of a non-metallic oxide layer. Through the combination of simulation analysis and experimental verification, it is concluded that increasing electrolyte pressure in stages can effectively improve machining accuracy and stability. The interstitial current increases as the feed rate of the tool electrode increases, and the diameter of the machined small hole decreases as it increases.

## 1. Introduction

High-temperature-resistant nickel-based alloy materials are widely used in the aerospace industry, in items such as working blades, turbine discs, and combustion chambers of aerospace engines, where GH4169 alloy is widely used in the manufacturing of aero engines in China. The specificity of the working environment requires the key structures to have high stability and reliability. The surface microscopic quality, machining residual stresses, and hardened layers generated during the machining process of critical components can have an important impact on their reliability. High-temperature-resistant nickel-based alloys generally work under certain stresses above 600 degrees Celsius. Therefore, they have high-temperature oxidation resistance and corrosion resistance. During processing, machining residual stresses, hardened layers, white layers, and other surface structures generated on the surface of high-temperature-resistant nickel-based alloys can have an impact on their stability and reliability [1,2,3,4,5]. The electrode position method was used to prepare the composite electrochemical coating on the surface of a nickel-based alloy. It can effectively improve the surface corrosion resistance and wear resistance [6,7,8].

Compared to transmission mechanical machining means, electrolytic machineries are more suitable for machining small hole structures on high-temperature-resistant nickel-based alloys. During electrolytic machining, there is no loss of electrodes, good quality of machined surfaces, no residual stresses, machining hardened layers, etc. [7,8,9,10,11,12,13,14]. Electrolytic machining has a high etching efficiency. Most importantly, the nature of the metal material has no effect on electrolytic processing and the equipment is simple. Hence, the method has received wide attention.

Many scholars have studied the performance of electrolytic machining on air mold cooling holes, process parameter control, multi-physics field simulation analysis of the machining gap, machining accuracy and machining efficiency [15,16,17,18]. Some scholars have conducted research in the area of the machining gap and feed control for electrolytic milling [19,20,21]. The influence of the internal flow field of the machining gap on the machining performance was analyzed by means of process tests and simulation analysis to optimize the servo control and thus improve the machining performance.

In this paper, the machining method and machining performance of small hole structures is studied by electrolytic machining of tube electrodes with high-temperature-resistant nickel-based alloy GH4169 as the machining object.

## 2. Performance Testing and Simulation Analysis of Electrolytic Processing Power Supply with Tube Electrode

**Figure 1:**
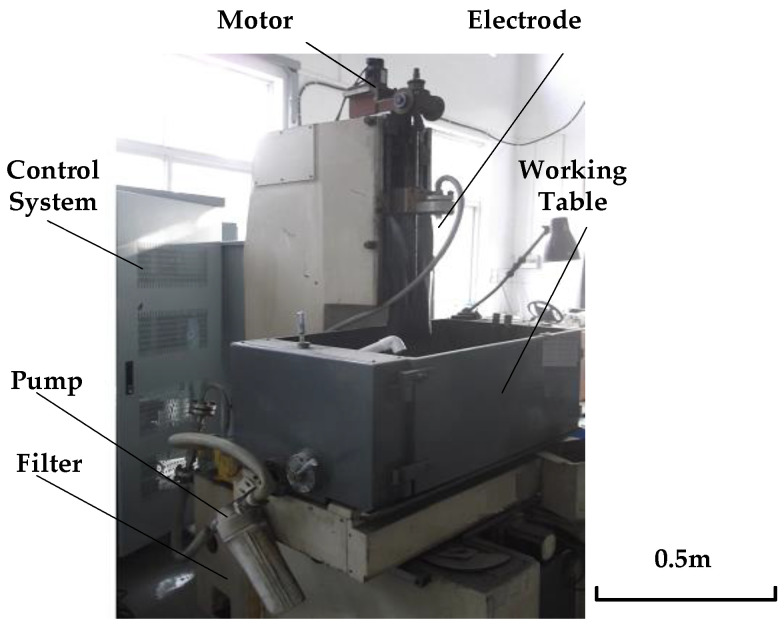
Diagram of ECM equipment processing small hole.

**Figure 2:**
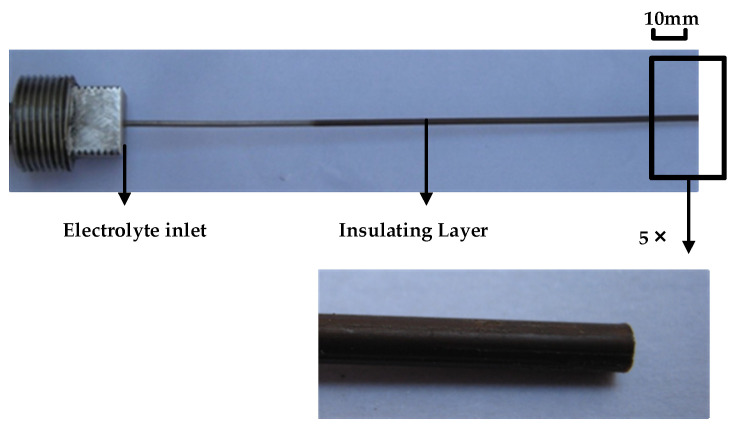
Titanium tube coated by tetrafluoroethylene.

The principle of electrolytic processing of small holes is shown in Figure 1. The metal titanium tube with insulating coating on the surface (shown in Figure 2) is connected to the negative pole of the power supply, and the machine tool spindle controls the tube electrode to move downward at a certain speed and uniformity. The anode workpiece is GH4169 alloy, which is connected to the positive side of the power supply. The high-speed flow of electrolytes plays the role of a conductive medium and updates the role of electrolytic heat and products. Under the action of the applied voltage, the anode metal is slowly etched away. The electrolysis products are carried away with the electrolyte flow until the shape of the workpiece is consistent with the shape of the workpiece surface. Therefore, the development of an appropriate power supply is an important guarantee for the stability and processing performance of electrolytic processing. This paper starts with a comparative analysis of the performance of DC and pulsed power supplies for electrolytic processing of tube electrodes.

Electrolytic processing is a complex process that uses electrochemical principles for processing. The process involves the interaction between physical fields such as electric field, flow field, heat conduction, chemical reaction, and structure. The electrolytic machining process of the tube electrode studied in this simulation mainly considers the effect of the coupling relationship between the electrolyte flow field and the electric field at the machining gap on the current distribution and sidewall removal rate at the anode surface. In order to simplify the model, the polarization phenomenon during the electrolytic processing is not considered. The effect of bubbles inside the gap on the electric field is neglected. The electrolyte conductivity is only subject to the variation depending on the electrolyte temperature. The fluid flow in the turbulent state of the electrolyte inside the processing gap is constrained by the conservation of momentum and is subject to the conservation of mass.

(1) $$\rho \left(u\cdot \nabla \right)u=\nabla \cdot \left[-\mathrm{pI}+\left(\mu +\mu_{T}\right)\left(\nabla_{u}+{\left(\nabla u\right)}^{T}\right)+F\right]$$

(2) ρ∇·(u)=0

In the equation: ρ is the fluid density; u is the fluid velocity; I is the current; t is the time; ∇ is the electrolyte dynamic viscosity; μ is the turbulent viscosity coefficient; T is the temperature of the electrolyte; and F is the external force acting on the fluid.

Based on incompressible fluids, the “turbulent, k-ε” interface solves the Navier–Stokes equation for momentum conservation as well as the continuity equation for mass conservation. The turbulence effect is modeled by a standard two-equation k-ε with a realizability constraints model to model them.

(3) $$\rho \left(u\cdot \nabla \right)k=\nabla \cdot \left[\left(\mu +\frac{\mu_{T}}{\sigma_{k}}\right)\nabla k\right]+P_{k}-\rho \varepsilon$$

(4) $$\rho \left(u\cdot \nabla \right)k=\nabla \cdot \left[\left(\mu +\frac{\mu_{T}}{\sigma_{\varepsilon }}\right)\nabla k\right]+C_{\varepsilon 1}\frac{\varepsilon }{k}\mathrm{pk}-C_{\varepsilon 2}\rho \frac{\varepsilon^{2}}{k}$$

In the equation: k is the turbulent kinetic energy; ε is the turbulent dissipation rate; σ_κ_ and σ_ε_ are the Prandtl values corresponding to the turbulent kinetic energy k and turbulent dissipation rate; and C_ε1_ as well as C_ε2_ are the model constants. According to the turbulence model parameters in COMSOL and test verification, k = 0.41, σ_κ_ = 1,σ_ε_ = 1.3, C_ε1_ = 1.44, and C_ε2_ = 1.92.

**Figure 3:**
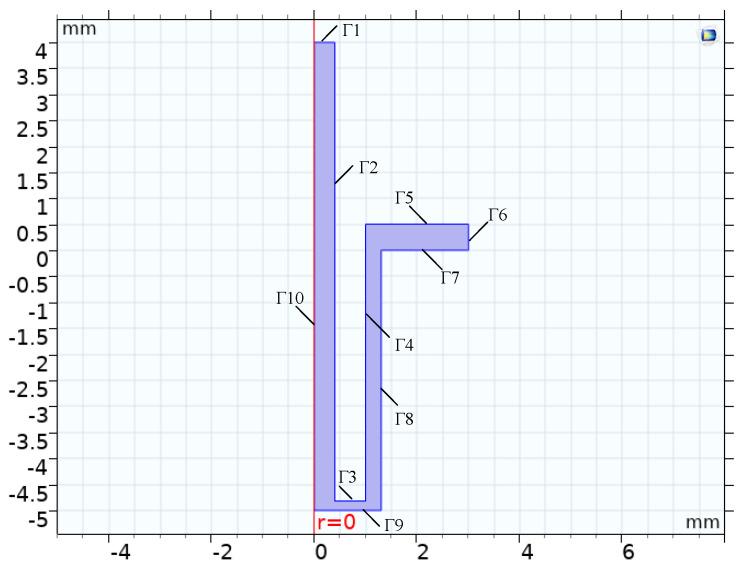
Two-dimensional model and boundary definition of machining area.

Simulation analysis is performed for the interactions between the flow, temperature, and electric fields in the tube electrode electrolytic processing gap. Figure 3 shows a schematic diagram of the two-dimensional asymmetric structure of the coupled multi-physics field simulation of the small hole in the electrolytic processing of the tube electrode. The solution area consists of the geometry of the cathode shape contour, the inlet and outlet, the anode workpiece machining contour, and the flow channel of electrolyte together. The boundaries Γ1 and Γ2 are the inner wall and front surface of the uncoated cathode. The boundary Γ2 is the outer wall of the cathode coated with an insulating layer. Boundaries Γ7, Γ8, and Γ9 are the workpiece anode, boundaries Γ1 and Γ6 are the inlet and outlet of the electrolyte, and boundary Γ5 is the wall.

**Figure 4:**
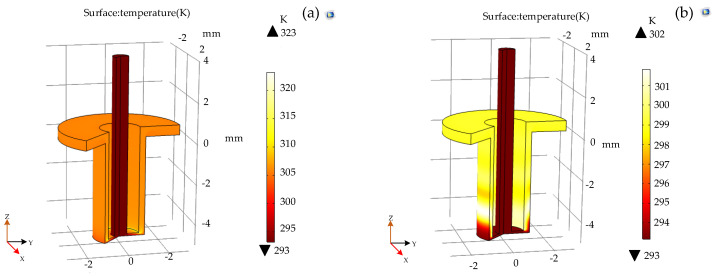
Temperature profile, (**a**)DC voltage and (**b**)pulse voltage.

**Figure 5:**
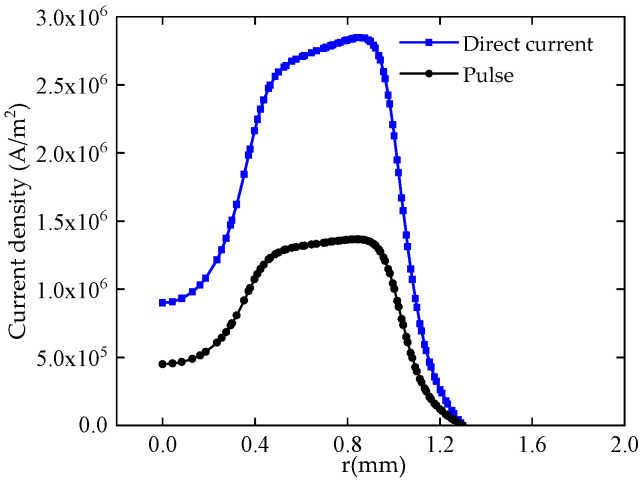
Machining gap current density distribution diagram.

The temperature distribution and current density distribution in the machining gap under the condition of external 24 V DC voltage and pulse voltage at both ends of the electrode and workpiece are shown in Figure 4 and Figure 5.

As can be seen in Figure 4, the sudden temperature change in the machining gap occurs at the connection between the end face of the electrode and the outer wall. The maximum temperature of the DC electrolytic process is 323 K at the equilibrium state, which is about 20 K higher than that of the pulsed electrolytic process, indicating that the pulsed electrolytic process can greatly improve the temperature distribution in the processing area and avoid the processing accuracy being affected by the high local temperature of the electrolyte.

Figure 5 shows that the current density on the workpiece surface increases and then decreases along the radius direction from the center of the tube electrode. The maximum value is about 1 mm radius, which is basically consistent with the temperature distribution in the machining gap. The maximum value of current density on the surface of the workpiece with DC voltage is 2.7 × 10^6^ A/m^2^, which is about 1.5 × 10 A/m^2^ higher than that of the pulsed voltage. The results show that pulse electrolysis can improve the current density distribution on the workpiece surface and reduce the short-circuiting problem caused by the uneven dissolution on the workpiece surface.

The advantage of a pulsed power supply is the periodic electrochemical anodic dissolution of the workpiece anode during processing. During the pulse interval, the power is intermittently depolarized, heat of reaction is transferred, and electrolysis products are discharged, effectively improving the problems of weak concentrated etching ability and large processing gap brought about by the use of a DC power supply, and improving the accuracy of electrolytic processing.

The frequency of short-circuiting increases as the tool electrode is used for a longer period of time. Since the workpiece material is a high-temperature-resistant nickel-based alloy containing non-metallic substances such as carbon and silicon, a large number of metal and non-metallic ions are oxidized at the anode to form ions with the following chemical equation:

M → M^+^ + e (5)

These ions enter the electrolyte of the processing gap and are subjected to strong electric and flow fields, which continuously impact and diffuse toward the end face of the tube electrode and are oxidized on the cathode surface. After adsorption and deposition, they form a non-metallic non-conductive film layer that weakens the electric field of the processing gap and causes a short circuit.

**Figure 6:**
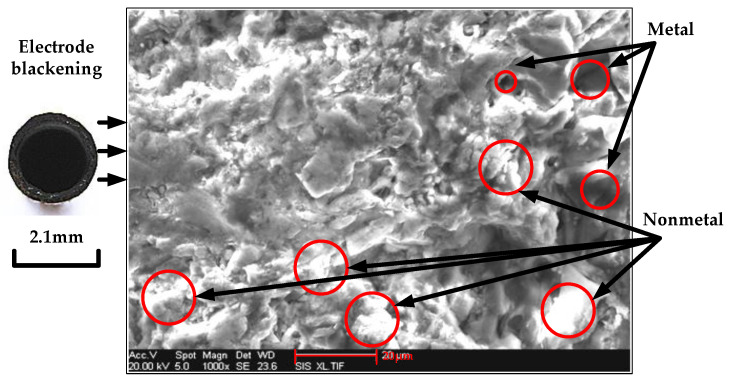
Micro morphology of tool electrode end face after short-circuiting.

**Figure 7:**
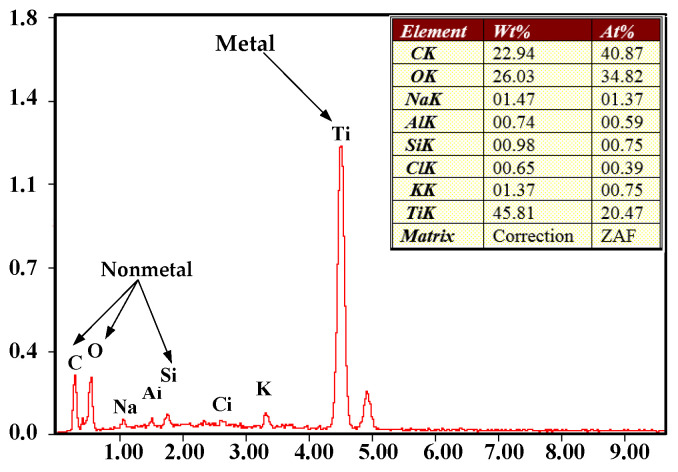
EDX spectrum of tool electrode end face after short-circuiting.

When a short circuit occurred, a scanning electron microscope of the FEISirion type from Philips was used to observe the morphology of the electrode end face. As shown in Figure 6, energy spectrum analysis was also performed to obtain the tissue composition of the tool electrode end face material, as shown in Figure 7.

**Figure 8:**
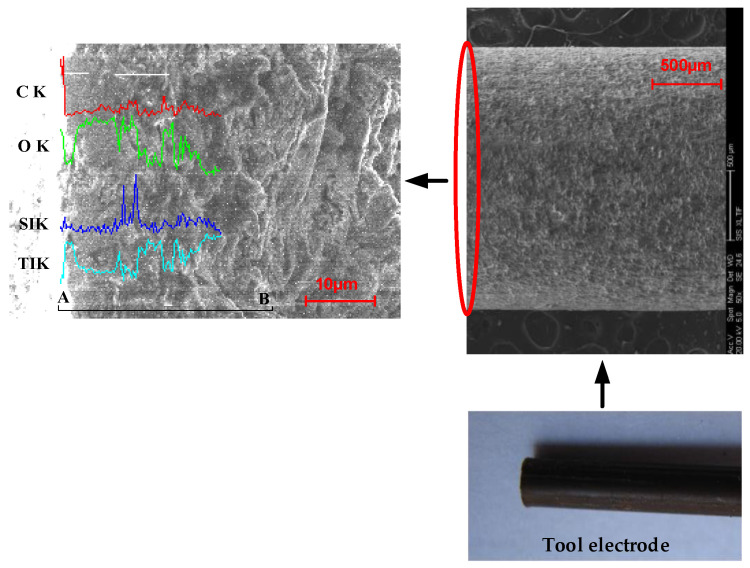
Sketch map of line scanning at A–B stage.

**Figure 9:**
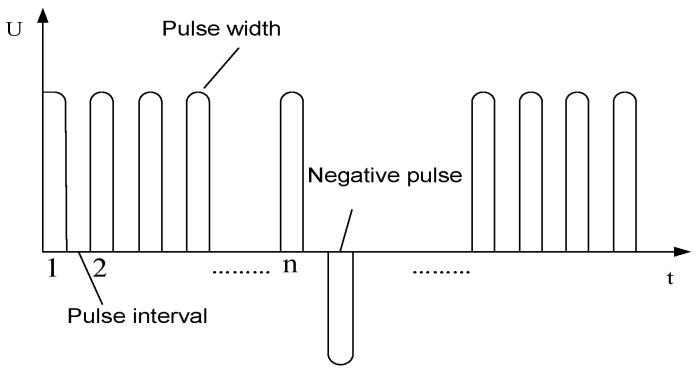
Structure diagram of pulse waveform.

From Figure 8, it can be seen that at the end face of the tube electrode, the three substances of carbon, oxygen, and silicon are very high and the content of titanium is relatively small. Along the outer wall of the tool electrode, they move from end A to end B. The content of three substances, carbon, oxygen, and silicon, decreases steeply, and the content of titanium increases and remains constant, which proves that the content of three sub-metallic substances, carbon, oxygen, and silicon, keeps increasing on the end face of the tool electrode as the electrolysis reaction proceeds, forming a surface film layer on the non-metallic particles on the end face of the tool electrode, which affects the electrolytic processing and thus causes a short circuit. Therefore, a high-frequency positive and negative pulse power supply is designed in this paper. Figure 9 shows the waveform schematic of a high-frequency positive and negative pulse power supply.

Appropriate negative pulses can remove non-metallic oxides from the electrode surface. Using alternate processing modes of positive and negative pulses, using positive pulses for workpiece electrolysis dissolution and negative pulses for non-metallic oxide removal can make electrolytic processing continue effectively and reduce the occurrence of short circuits.

## 3. Simulation Analysis of Flow Field of Electrolytic Processing of High-Frequency Positive and Negative Pulse Tube Electrodes

The magnitude of the flow field in the processing gap affects the electrolytic etching products, processing heat. Therefore, COMSOL finite element simulation software was used to simulate the flow field of the process of electrolytic processing of small holes with tube electrodes. In the turbulent physical field, the initial boundary conditions are as follows: the initial inlet pressure of the electrolyte is 2.1 MPa and the outlet pressure is atmospheric pressure. The inlet pressure of electrolyte is increased by 0.3 MPa for every 5 mm increase in processing depth. The temperature of electrolyte is 293.15 K in ideal conditions, the dynamic viscosity of electrolyte is 1.01 × 10^−3^ Pa.s, and the density is 1.0 × 10^3^ Kg/m^3^.

### 3.1. Electrolyte Flow Field Distribution under Constant Pressure

**Figure 10:**
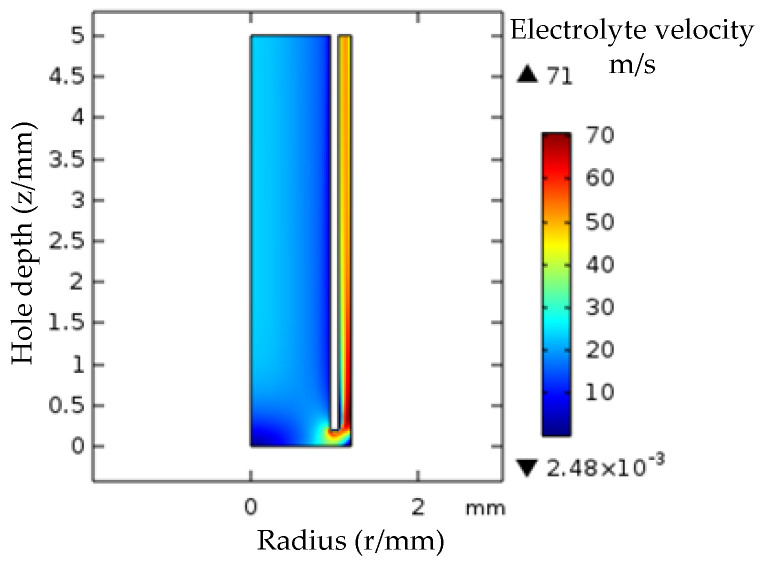
Cloud diagram of electrolyte velocity distribution.

When the electrolyte inlet pressure is 2.1 MPa and the processing depth is 5 mm, the velocity distribution cloud of the flow field in the processing gap is shown in Figure 10. The electrolyte flow velocity variation interval inside the tube electrode and at the electrolyte outlet is not large, and the flow field is relatively uniform. The main area of flow field variation is located in the front gap between the tube electrode and the bottom of the small hole, and the low velocity area of electrolyte flow is concentrated in the center of the bottom of the small hole.

**Figure 11:**
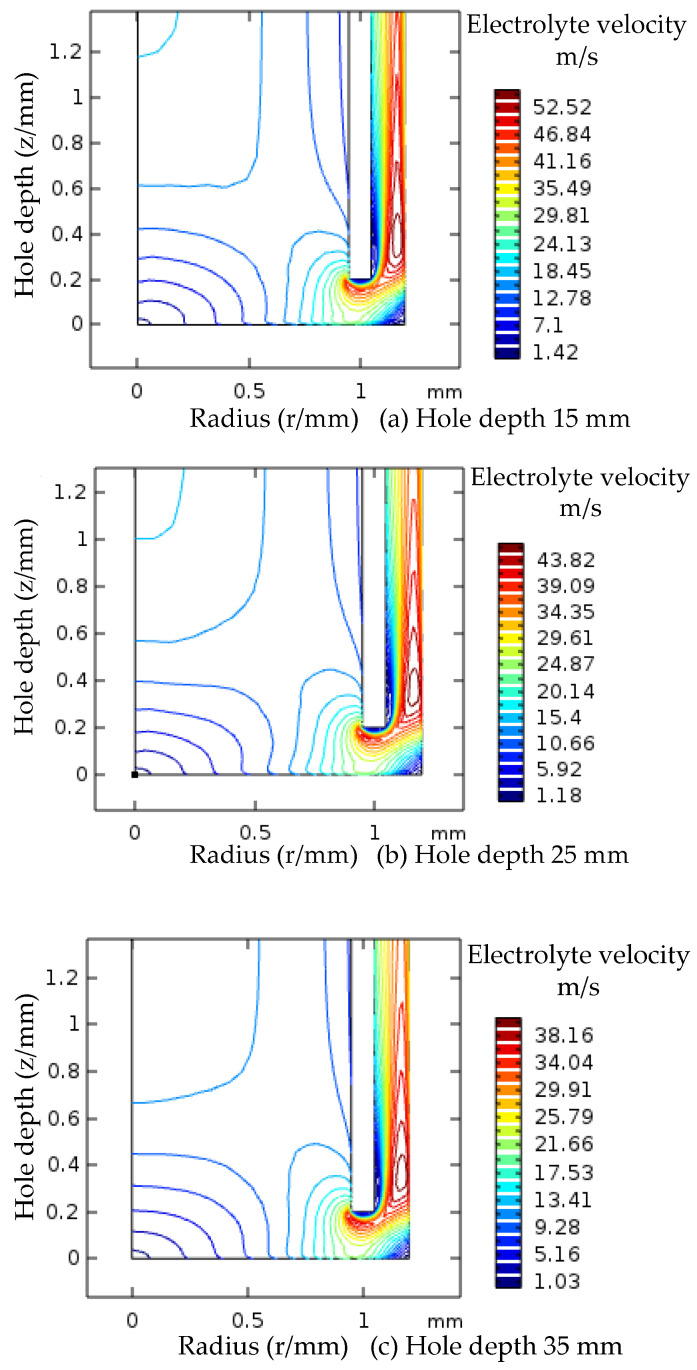
Velocity contour map of electrolyte low-speed region in the machining gap.

During processing, the low velocity flow of electrolyte is not conducive to the discharge of electrolytic products and heat in the processing gap, which affects the stability of electrolytic processing. The contour graph of electrolyte in the gap indicates the change of flow rate magnitude in the processing area. The velocity contour diagram of the low velocity zone of electrolyte is shown in Figure 11 when the inlet pressure of electrolyte is 2.1 MPa and the processing depth of the small hole is different. The velocity of the electrolyte in the gap decreases with the increase of the processing depth, and the range of the low velocity zone contour increases gradually. The range of velocity contour values in the same low-speed zone is as follows: hole depth 15 mm, contour number 1.18–10.66 m/s; hole depth 35 mm, contour value 1.03–9.28 m/s.

The electrolyte inlet pressure is 2.1 MPa. The trend of electrolyte flow rate change is basically the same when the processing depth increases, and the flow rate difference at the same reference point gradually increases along the radius direction of the low-speed zone.

### 3.2. Electrolyte Flow Field Distribution under Booster Pressure

**Figure 12:**
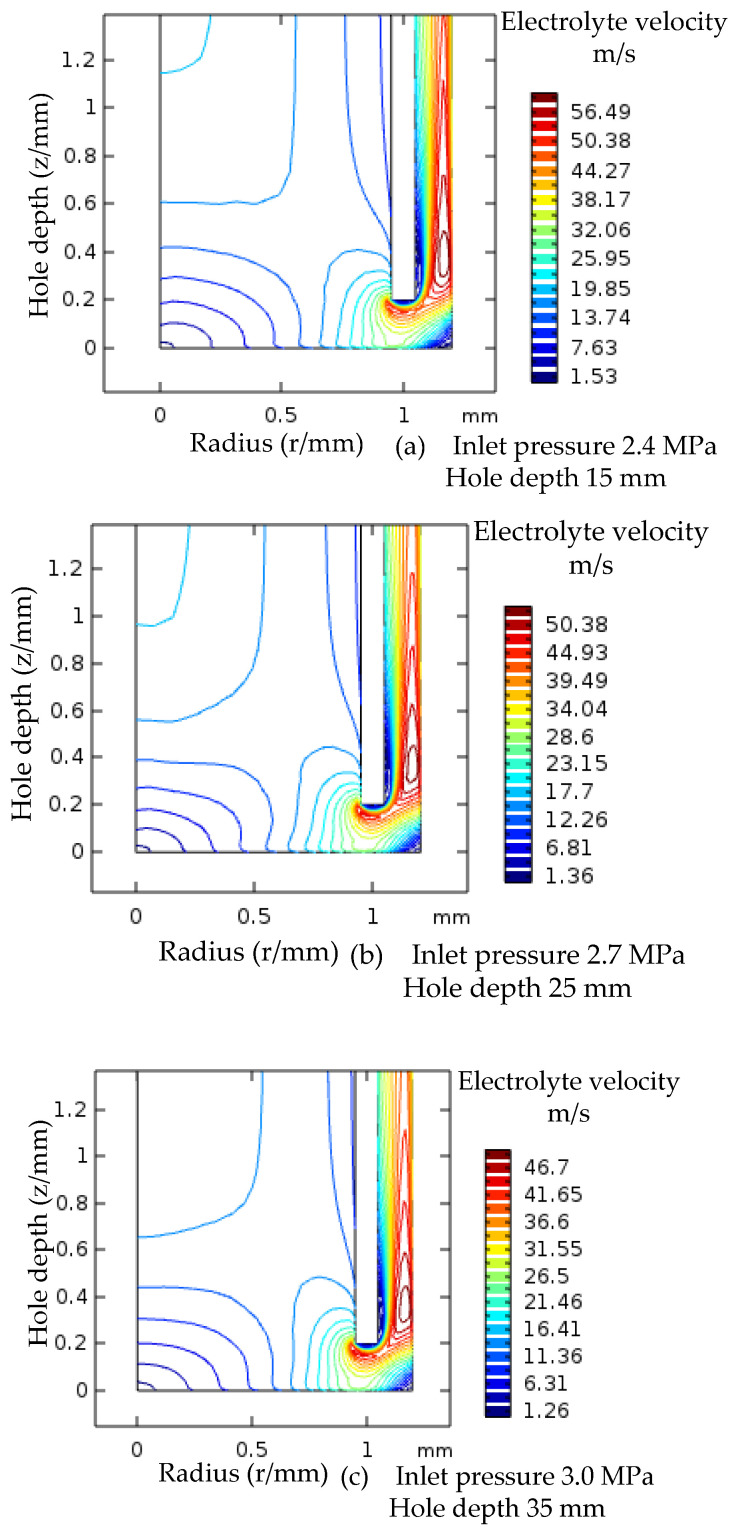
Velocity contour map of the low-speed region after boosting.

The electrolyte flow rate in the gap decreases with the increase of machining depth. Therefore, in the simulation experiment, the analysis of machining depth and electrolyte pressure variation was carried out, and the contour plot of electrolyte flow rate in the low-speed zone for a 5 mm increase in machining depth and a 0.3 MPa increase in electrolyte pressure is shown in Figure 12. The gap between the contour values in the low-speed zone becomes smaller, and the electrolyte flow rate fluctuates within a certain range.

The electrolyte flow rate is greatly enhanced by increasing the electrolyte pressure. The difference in flow rate at the reference point is greatly reduced for small hole processing depths of 5 mm, 15 mm and 25 mm. The flow rate is more stable during the whole electrolytic processing.

To sum up, in the process of small hole electrolytic processing, the low-speed zone in the flow field of the processing gap is greatly reduced after increasing the electrolyte pressure in a segmented manner, and the flow speed of the electrolyte increases, which can take away the electrolytic products and Joule heat in the processing gap in a timely manner, improving the stability and fixed domain of electrolytic processing.

## 4. Experimental Study on Electrolytic Processing of High Frequency Positive and Negative Pulse Tube Electrodes

In the experiment, the workpiece material is GH4196 alloy, the tool electrode is a titanium tube with 1.8 mm inner diameter and 2.1 mm outer diameter, the outer insulation layer is applied, the electrolyte is nitric acid solution, the initial machining gap is 1 mm, and the method of an orthogonal test is used. A 24 V pulse power supply was adopted. The electrolyte was a nitric acid solution, and the electrolyte pressure was 1.8–3.0 MPa. The feeding speed of the electrode tool was 0.72–1.2 mm/min, and the side of the electrode tool was covered with an insulating layer. The electrolytic reaction occurred only at the front end of the electrode tool. The insulation material was PTFE with a thickness of approximately 0.1 mm and a surface resistivity >1 × 10^10^ Ω.

The correlation between the variables and the corresponding regression equation were determined according to the data samples. According to five independent parameters, namely, pulse width *T*_on_, duty cycle *D*, electrolyte pressure *P*, electrolyte concentration *C*, and electrode tool feeding speed *V*, atest schedule was established, as shown in Table 1.

According to the experimental data given in Table 1 and through the experimental analysis of regression orthogonal combinations, the mathematical model between the evaluation criteria and process parameters was established, and the equation is as follows:

The unilateral clearance on the side of the hole is the radial cutting value along the machining direction, which can be expressed as:

(6) $$g_{s}=\frac{1}{2n}\overset{n}{\underset{i=1}{\sum }}\left(r_{1}-r_{2}\right)$$

where

n—The number of positions the aperture has measured along the depth direction

r_1_—Machining hole diameter (mm)

r_2_—Tool electrode outside diameter (mm)

The linear removal rate is the electrochemical machining depth in unit time, which can be expressed as:

(7) $$L_{S}=\frac{m}{\rho \times \mathrm{At}}$$

where

ρ—Workpiece material density (kg/m^3^)

A—Average hole area (m^2^)

m—Total removal mass (kg)

t—Processing time (min)

According to the experimental data given in Table 1, the regression orthogonal combination experiment was used for the analysis. Finally, the mathematical model between the evaluation criteria and process parameters is established. The equation is as follows:

(8) $$y=b_{0}+{\sum_{1}^{n}b}_{i}x_{i}+{\sum_{1}^{n}b}_{ii}x^{2}{}_{ii}+{\sum_{i<j}^{}b}_{i}x_{j}$$

where

y—Amount of response of the research (*g*_s_/mm)

*b_i_b_ii_b_ij_*—Coefficients of regression

*b*_0_—Constant

*x*_1_—Process variable (*T*_on_/μs)

*x*_2_—Process variable (*D*/%)

*x*_3_—Process variable (*P*/MPa)

*x*_4_—Process variable (*C*/%)

*x*_5_—Process variable (*V*/mm/min)

The values of actual test parameters and evaluation parameters obtained through the orthogonal experiment are shown in Table 1.Then, the scientific analysis is conducted by utilizing SPSS software. Equation (8) can be calculated to obtain the equation coefficient used to evaluate the one-side clearance of the hole side. It is used to evaluate the unilateral clearance on the side of the hole, as shown in Table 2.

The analysis results show that the value of *F*_0_ obtained by the single-side gap model is greater than *F*_0.05_(16,11) = 2.96. Therefore, for a given significant level α = 0.05, excluding *C*, (*TP*), (*TV*), and (*CV*), parameters *T*_on_, *D*, *P*, *V*, and all the mutual variables have significant effects. In addition to the process variables with little influence in the regression coefficient, the regression equation of the one-sided gap at the side of the hole is as follows:

(9) $$y=-0.112+0.00061T_{\mathrm{on}}-0.042D+0.799P+0.026V-0.0000013T_{\mathrm{on}}D-\;0.000017T_{\mathrm{on}}C+0.0027DP-0.000014DC+0.0122CV-0.0184PC-1.03PV+\;0.024P^{2}+0.009C^{2}y$$

In order to verify the accuracy of the model, repeated tests and selected parameters are shown in Table 3.

The true value, predicted value, and error rate of the single-side clearance of the hole are shown in Table 4.

**Figure 13:**
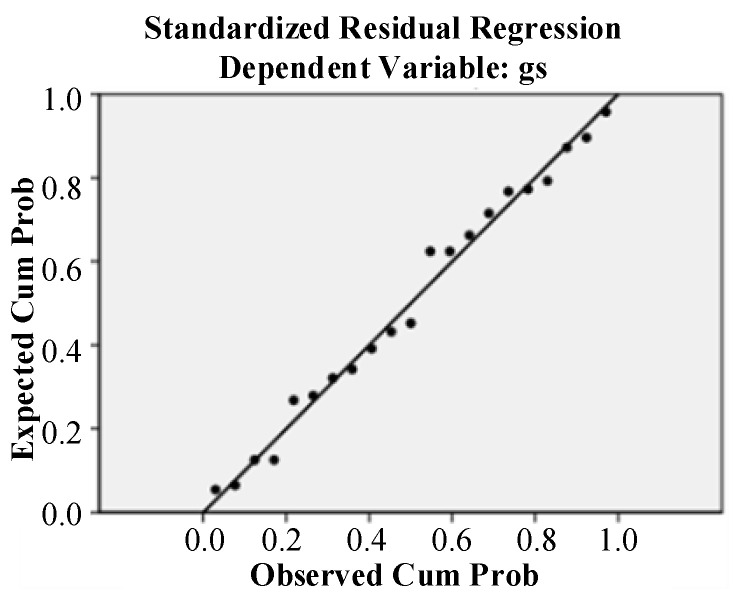
Normal P–P plot between the observed cumulative probability and expected cumulative probability.

Additionally, the normal P–P graph between the cumulative probability of parameter variables and the cumulative probability of the model-predicted values was analyzed. As shown in Figure 13, the scatter points showed a linear trend, and no extreme values were found.

According to the analysis process of the variables in the regression equation of the hole side gap, the unimportant variables in the model are removed. The linear removal rate regression equation was obtained in detail as shown in Table 5.

The regression equation of linear removal rate is as follows:

(10) $$y_{1}=3.33-0.0029T_{\mathrm{on}}-0.82P+0.0011T_{\mathrm{on}}P-0.0089P^{2}-0.089V^{2}$$

**Figure 14:**
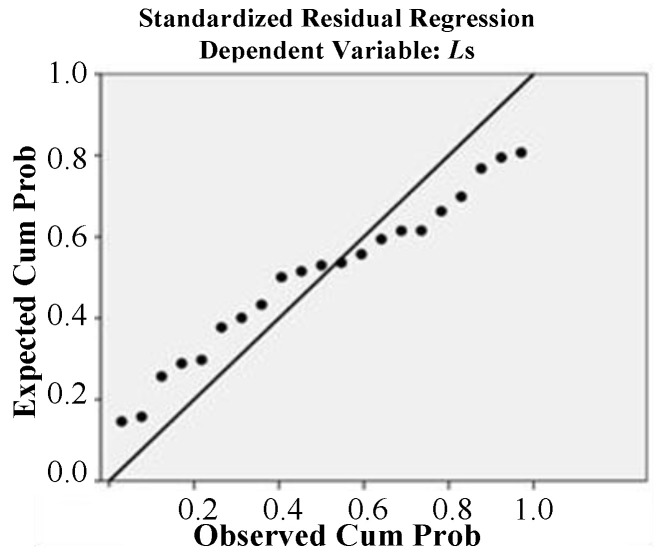
Normal P–P plot between observed cum prob and expected cum prob of *L*s.

Additionally, the normal P–P graph between the cumulative probability of parameter variables and the cumulative probability of the model-predicted values wasanalyzed. As shown in Figure 14, the scatter points showed a linear trend, and no extreme values were found.

Similarly, in order to verify the accuracy of the model, it conducts repeated tests.The parameters are selected as shown in Table 3.The true and predicted values of the linear removal rate and the error rate are shown in Table 6.

The results show that there is little difference between the real value and the predicted value of linear removal rate. The prediction of linear removal rate by Equation (10) is real and reliable.

**Figure 15:**
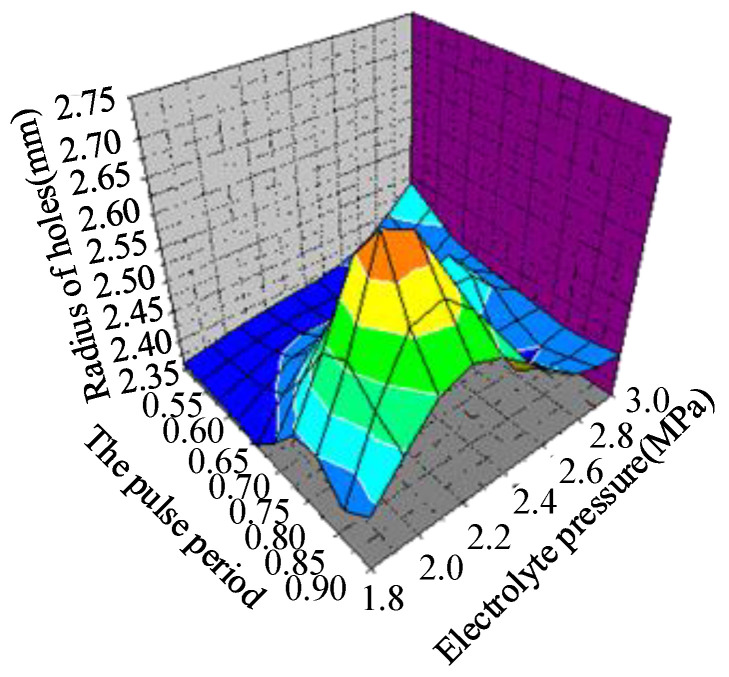
Response surface diagram of the entrance diameter.

Figure 15 shows that when the working cycle and electrolyte pressure increase, the diameter of the small deep hole increases, and the distribution is in a normal curve. When the electrolyte pressure was 2.4–2.6 MPa, and the working cycle was 0.75–0.80, the entrance diameter reached the maximum value.

**Figure 16:**
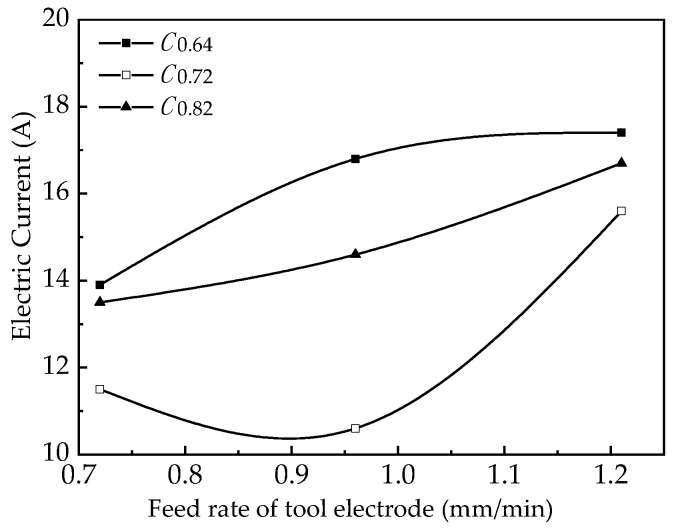
Electric-current sampling of ECM.

**Figure 17:**
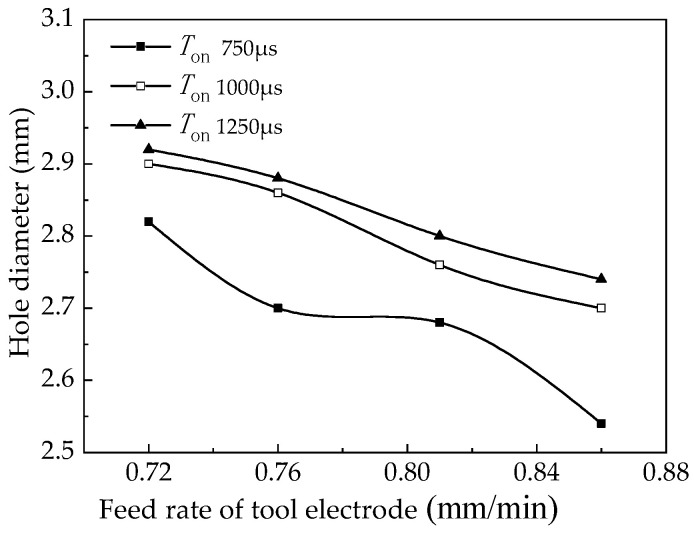
Influence of feed rate of tool electrode on the diameter of processed hole.

At the same time, the machining current during the machining process was collected as shown in Figure 16. The feed rate of tool electrode versus hole diameter is shown in Figure 17.

The analysis shows that the gap current increases with the increase of the feed rate of the tool electrode at different working cycles, the front-end machining gap decreases, the potential gradient between the tool electrode and the workpiece increases, and the current density increases. As the feed rate of the tool electrode increases, the linear removal rate of small holes also increases, which in turn improves the fixed domain of small-hole machining and the diameter of small holes decreases with it.

## 5. Conclusions

1. The simulation analysis compares the tube electrode electrochemical machining under the action of DC power supply and pulse power supply. Pulse electrochemical machining can greatly improve the temperature distribution in the machining area.It improves the current density distribution on the workpiece surface, improves the machining accuracy and avoids short-circuiting problems.
2. Scanning electron microscopy was used to analyze the microscopic morphology and composition of the electrode end face during short-circuiting. When short-circuiting, a non-metallic film layer is formed on the electrode end face. Its main non-metallic components are C, O, and Si, and affects the processing stability.
3. The gap flow field in ECM of high-frequency positive and negative pulse power supply tube electrode was analyzed. The flow field in the machining clearance changes with different machining depth. The piecewise method to increase the electrolyte pressure is used to reduce the low-speed zone of the flow field in the machining clearance. In the processing gap, the electrolytic product, heat renewal speed, processing accuracy and localization are improved.
4. Through experimental analysis, the mathematical model between the hole side gap and five technological parameters was established. Through linear regression analysis of variance, we can see that the interaction variables have significant influence. The error between the actual value and the predicted value of the hole side gap is very small. It can be accurately evaluated by the mathematical model of the hole side gap.

## Figures and Tables

**Table 1:** Experimental conditions and evaluation parameters.

No.	*T*_on_ (μs)	*D* (%)	*P* (MPa)	*C* (%)	*V* (mm/min)	*g*_s_ (mm)	*L*_s_ (mm/min)
1	1000	0.82	2.7	7	0.96	0.10	0.96
2	500	0.72	2.4	7	1.20	0.09	1.04
3	500	0.72	2.7	10	0.96	0.27	0.96
4	250	0.56	3.0	13	0.72	0.20	0.73
5	250	0.64	2.4	20	1.08	0.15	1.08
6	1250	0.82	3.0	15	0.84	0.26	0.83
7	1000	0.82	2.1	20	0.72	0.34	0.72
8	750	0.72	1.8	20	1.08	0.14	1.10
9	750	0.72	2.7	15	0.84	0.18	0.84
10	500	0.72	3.0	7	0.84	0.28	0.83
11	750	0.82	2.1	13	1.20	0.35	1.21
12	250	0.64	1.8	15	0.84	0.43	0.84
13	250	0.64	2.1	15	0.96	0.48	0.96
14	1000	0.72	3.0	15	1.08	0.20	1.07
15	750	0.72	2.4	10	0.72	0.24	0.71
16	1250	0.82	2.4	7	0.96	0.24	0.96
17	500	0.64	2.7	7	0.72	0.16	0.73
18	1000	0.72	1.8	13	1.20	0.10	1.20
19	1250	0.82	2.7	13	1.08	0.16	1.09
20	1250	0.82	3.0	10	1.20	0.06	1.19
21	1250	0.86	1.8	13	0.72	0.15	0.71
22	500	0.64	2.1	20	1.08	0.15	1.08
23	250	0.64	2.7	10	1.20	0.08	1.22
24	750	0.81	3.0	20	0.96	0.24	0.93
25	1000	0.72	2.4	10	0.72	0.31	0.71

**Table 2:** Regression analysis and ANOVA for $g_{s}$.

Model	Coefficients	Sig.	Model	Coefficients	Sig.
Constant	−0.112	0.000	*x*_2_ *x*_5_	0.0122	0.042
*x*_1_	0.00061	0.026	*x*_3_ *x*_4_	−0.0184	0.018
*x*_2_	−0.042	0.009	*x*_3_ *x*_5_	−1.03	0.023
*x*_3_	0.799	0.039	*x*_3_ *x*_3_	0.024	0.016
*x*_5_	0.026	0.015	*x*_4_ *x*_4_	0.009	0.067
*x*_1_ *x*_2_	−0.0000013	0.024			
*x*_1_ *x*_4_	−0.000017	0.021			
*x*_2_ *x*_3_	0.0027	0.043			
*x*_2_ *x*_4_	−0.000014	0.011			
ANOVA	df	SS	MS	F_0_	Sig.
Regression	16	0.501	0.026	12.30	0.002
Residual	4	0.011	0.0013		
Total	20	0.512			

**Table 3:** Validation experiments.

No.	*T*_on_ (μs)	*D*	*P* (MPa)	*C* (%)	*V* (mm/min)
1	500	0.72	3.0	7	0.84
2	750	0.82	2.1	13	1.20

**Table 4:** Percentage error of *g*_s_.

Measured Value (mm)	Predicted Value (mm)	Error (%)
0.28	0.22	21.42
0.35	0.34	2.86

**Table 5:** Regression analysis and ANOVA for $L_{s}$.

Model	Coefficients	Sig.	Model	Coefficients	Sig.
Constant	3.33	0.000	*x*_3_ *x*_3_	−0.089	0.097
*x*_1_	−0.0029	0.000	*x*_5_ *x*_5_	−0.089	0.053
*x*_3_	−0.82	0.071			
*x*_1_ *x*_3_	0.0011	0.050			
ANOVA	df	SS	MS	F0	Sig.
Regression	19	0.59	0.031	12.25	0.004
Residual	1	0.003	0.003		
Total	20	0.59			

**Table 6:** Percentage error of *L*_s_.

Measured Value (mm)	Predicted Value (mm)	Error (%)
0.83	0.93	1.20
0.96	0.99	3.75

## Data Availability

Data are contained within the article.

## Abbreviations

SEM	Scanning electron microscope
CD	Direct current
MPa	Million pascal
ECM	Electrochemical machining
*T*_on_	Pulse width
D	Working cycle
P	Electrolytic hydraulic pressure
C	Concentration
PTFE	Polytetrafluoroethylene
